# Attenuating bone loss in osteoporosis: the potential of corylin (CL) as a therapeutic agent

**DOI:** 10.18632/aging.205885

**Published:** 2024-06-11

**Authors:** Song Zhou, Junming Huang, Kun Chen, Qixuan Wang, Zheng Liu, Yanli Sun, Feng Yin, Shanjin Wang, Zhiying Pang, Min Ma

**Affiliations:** 1 Department of Joint Surgery, Shanghai East Hospital, School of Medicine, Tongji University, Shanghai 200092, China; 2 The Orthopedic Hospital, The First Affiliated Hospital, Jiangxi Medical College, Nanchang University, Nanchang, Jiangxi 330006, China; 3 Department of Sports Medicine, Orthopedic Hospital, The First Affiliated Hospital, Jiangxi Medical College, Nanchang University, Nanchang, Jiangxi 330006, China; 4 Department of Orthopedics, The First Affiliated Hospital of USTC, Division of Life Sciences and Medicine, University of Science and Technology of China, Hefei, Anhui 230001, China; 5 Department of Anesthesiology, Shanghai East Hospital, School of Medicine, Tongji University, Shanghai 200092, China; 6 Shanghai Institute of Stem Cell Research and Clinical Translation, Shanghai 200120, China; 7 Department of Spine Surgery, Shanghai East Hospital, School of Medicine, Tongji University, Shanghai 200092, China

**Keywords:** corylin, osteoporosis, osteoclast, mitochondria, RANKL

## Abstract

The global prevalence of osteoporosis is being exacerbated by the increasing number of aging societies and longer life expectancies. In response, numerous drugs have been developed in recent years to mitigate bone resorption and enhance bone density. Nonetheless, the efficacy and safety of these pharmaceutical interventions remain constrained. Corylin (CL), a naturally occurring compound derived from the anti-osteoporosis plant *Psoralea corylifolia* L., has exhibited promising potential in impeding osteoclast differentiation. This study aims to evaluate the effect and molecular mechanisms of CL regulating osteoclast differentiation *in vitro* and its potential as a therapeutic agent for osteoporosis treatment *in vivo*. Our investigation revealed that CL effectively inhibits osteoclast formation and their bone resorption capacity by downregulating the transcription factors NFATc1 and c-fos, consequently resulting in the downregulation of genes associated with bone resorption. Furthermore, it has been observed that CL can effectively mitigate the migration and fusion of pre-osteoclast, while also attenuating the activation of mitochondrial mass and function. The results obtained from an *in vivo* study have demonstrated that CL is capable of attenuating the bone loss induced by ovariectomy (OVX). Based on these significant findings, it is proposed that CL exhibits considerable potential as a novel drug strategy for inhibiting osteoclast differentiation, thereby offering a promising approach for the treatment of osteoporosis.

## INTRODUCTION

Osteoporosis is a prevalent systemic metabolic disorder observed primarily in the elderly population, distinguished by the deterioration of bone density and compromised bone microstructure, ultimately resulting in heightened vulnerability to bone fragility and susceptibility to fractures. Based on statistical data, the global prevalence of osteoporosis exceeds 200 million individuals [1]. As individuals age, the incidence of osteoporosis escalates, positioning it as the seventh most prevalent ailment. Given its high prevalence, severe cases of osteoporosis can lead to systemic harm, prompting the World Health Organization (WHO) to classify it as one of the three primary diseases affecting middle-aged and elderly populations, thereby designating it a global public health concern [2]. Since humans are living longer, osteoporosis has become an important health issue, thus the prevention and treatment of this disease are of significant importance.

Among the tissues in the human body, bone undergoes the most dynamic changes. During growth and development, bone shape and integrity are maintained by bone remolding, in which broken bone is dissolved and absorbed by osteoclasts, and the remaining fractures or injuries in the bone are filled by osteoblasts that differentiate and develop new bone tissues. In the course of advancing age and hormonal cessation, the process of healthy bone remodeling gradually becomes disrupted. Osteoclasts, the exclusive cells accountable for bone resorption, demonstrate deviations in both their quantity and functionality, ultimately resulting in the occurrence of bone loss [3]. Therefore, osteoclasts are widely recognized as a significant focus of research for the development of anti-osteoporosis medications. Over the past two decades, a range of anti-osteoclastogenic medications targeting osteoclasts have been employed in clinical settings, including bisphosphonates, selective estrogen receptor modulators, and RANKL monoclonal antibodies. Despite the abundance of anti-osteoporotic drugs available, their therapeutic outcomes have not met the desired standards. Long-term use of bisphosphonates has been associated with complications such as osteonecrosis of the jaw and nonspecific femur fractures [4]. Selective estrogen receptor modulators have been known to induce painful spasms in the lower limbs, venous thrombosis, and even severe strokes [5]. Similarly, RANKL monoclonal antibody exhibits a specific impact on antiresorptive effects, but extended utilization has been associated with heightened susceptibility to cutaneous eruptions, infections, atypical femur fractures, and osteonecrosis of the jaws, which lead to further substantiation about the safety profile of this therapeutic agent [6]. As a result, the quest for efficacious osteoporosis treatments devoid of significant adverse reactions remains an imperative undertaking.

In recent years, the escalating time and cost associated with research and development of chemically synthesized drugs have prompted researchers to turn their attention towards natural drug monomers. This is primarily due to the favorable attributes exhibited by these compounds, including low toxicity, natural synthesis, and a diverse array of pharmacological effects. Consequently, Natural Compounds (NC) have garnered significant interest among a multitude of researchers. Numerous studies have previously substantiated the effectiveness of natural compounds in addressing pathological obesity, diabetes, cardiovascular disease, and related conditions [7]. Our previous investigations have likewise corroborated the favorable therapeutic outcomes of natural compounds in bone metabolic disorders through their impact on osteoclasts [8]. Furthermore, abundant natural resources in China and extensive heritage in traditional Chinese medicine confer a distinctive advantage for conducting research on natural compounds.

Herb *Psoralea corylifolia* L., also called “buguzhi” in Chinese, has been extensively used in Chinese medicine to treat osteoporosis for centuries. Among the natural products within *Psoralea corylifolia* L., a representative flavonoid compound corylin possesses multiple biological activities, such as anti-inflammatory properties [9, 10], regulating antioxidant activity [11], and suppressing tumor progression [12, 13]. In bone metabolism study, corylin proved to be a successful element in displaying osteoblastic proliferative activities and osteogenic effects [14]. In addition, a recent study reported that corylin inhibits osteoclast formation by using bone marrow macrophages [15]. However, the evidence regarding the effect of corylin on intracellular signaling pathways during osteoclast differentiation and therapeutic efficacy of corylin on osteoporosis is still limited. In this study, we aimed to investigate the effects and mechanisms of corylin on osteoclast induced from bone marrow macrophage (BMMs) *in vitro* and bone loss induced by ovariectomy *in vivo*.

## MATERIALS AND METHODS

### Reagents

Except for those specifically indicated, all reagents used in cell culture were purchased from Thermo Fisher Scientific (Waltham, MA, USA). Recombinant Murine RANKL and M-CSF were obtained from R&D systems (Minneapolis, MN, USA) and were dissolved in 0.1% BSA to create a stocking solution with a concentration of 10 ng/μl. Corylin (CL; C_20_H_16_O_4_; MW: 320.34) with a purity of 99.97% was produced by MedChemExpress (Shanghai, China) and were dissolved in 0.1% BSA to create a stocking solution.

### Cells culture

Following the previously described method with minor modifications [3, 16], we isolated the whole bone marrow cells of long bones (tibias and femurs) from 6-week-old mice. They were then cultured in complete α-MEM (Gibco, 22561-021) with 30 ng/ml M-CSF (R&D Systems, 416-ML-050) supplemented with 10% fetal bovine serum (Gibico, 10100147) and 1% Penicillin-Streptomycin (Gibico, 15140122) at 37°C for 3 days and the final adherent cells were bone marrow macrophages (BMMs).

### Cell viability assay

BMMs were seeded into 96-well plates with bone marrow macrophage complete media and cultured for 24 hours. Following that, the BMMs were treated with DMSO and different doses of CL for indicated days. Using the CCK-8 commercial kit, the cytotoxicity of CL on BMMs was detected by a microplate reader at 450 nm.

### Osteoclast differentiation and tartrate resistant acid phosphatase (TRAP) staining

BMMs were seeded into 96-well plates with bone marrow macrophage complete media and culture for 24 hours. Following that, the BMMs were cultured with osteoclast induction media (MEM-α complete medium containing 30 ng/mL M-CSF and 50 ng/mL RANKL). During osteoclast differentiation, induction media was changed every two days and the TRAP staining Kit was used to label formed osteoclast. In captured photo of osteoclast, TRAP positive with multiple nuclei (>3 nuclei) were defined as mature osteoclast.

### F-actin ring assay

BMMs were seeded onto the bone plate (Corning Incorporated Life Science, NY, USA) and osteoclasts were induced as above mentioned. For the quantification of osteoclasts formation, fixation with 4% paraformaldehyde (PFA) and permeabilization with 0.1% Triton X-100 (v/v) followed by staining with Actin-Tracker and DAPI were performed. The fluorescence microscope (Olympus IX-71) was used to explore osteoclastic F-actin ring images.

### Bone resorption assay

The differentiated osteoclasts were induced by the above method and then reseeded on bone plate (Corning Incorporated Life Science, NY, USA). Following 3 days of culture, 5% sodium hypochlorite was used to remove osteoclasts and the resorption pits in bone plate were detected.

### RNA isolation and quantitative RT-PCR

Following the previously described method with minor modifications [17], BMMs were cultured with osteoclast induction media. During osteoclast differentiation, DMSO or CL was supplemented in osteoclast induction media. As directed by the manufacturer, total RNA from cells cultured for 3 days was extracted by RNA-Quick Purification Kit (YISHAN Bio-Tec, Shanghai, China) and reverse transcription into cDNA by Prime Script RT reagent kit (TaKaRa Biotechnology, Kusatsu, Japan). As templates, reverse-transcript cDNA was used for qRT-PCR using SYBR (Vazyme, Nanjing, China). For normalizing relative expression, GAPDH was chosen as a housekeeping gene and the specific primers for individual markers were listed in Table 1.

**Table 1:** List of primers used in quantitative real-time RT-PCR.

**Target gene**	**Sense sequence (5′ to 3′)**	**Antisense sequence (5′ to 3′)**
NFATc1	GACCCGGAGTTCGACTTCG	TGACACTAGGGGACACATAACTG
c-fos	CGGGTTTCAACGCCGACTA	TTGGCACTAGAGACGGACAGA
Cathepsin K	GAAGAAGACTCACCAGAAGCAG	TCCAGGTTATGGGCAGAGATT
MMP9	CTGGACAGCCAGACACTAAAG	CTCGCGGCAAGTCTTCAGAG
RANK	GGACGGTGTTGCAGCAGAT	GCAGTCTGAGTTCCAGTGGTA
TRAP	CACTCCCACCCTGAGATTTGT	CATCGTCTGCACGGTTCTG
DC-STAMP	GGGGACTTATGTGTTTCCACG	ACAAAGCAACAGACTCCCAAAT
OC-STAMP	CTGTAACGAACTACTGACCCAGC	CCCAGGCTTAGGAAGACGAAG
ATPv0d2	CAGAGCTGTACTTCAATGTGGAC	AGGTCTCACACTGCACTAGGT
GAPDH	AGGTCGGTGTGAACGGATTTG	TGTAGACCATGTAGTTGAGGTCA

### Western blot assay

Following the previously described method with minor modifications [16], BMMs were cultured with osteoclast induction media. Total proteins were extracted using RIPA lysis buffer supplemented with broad spectrum phosphatase inhibitors and phenylmethylsulfonyl fluoride (PMSF). Quantification was performed by using BCA protein assay kit (Thermo Fisher Scientific, Waltham, MA, USA). Afterwards, 20 μg prepared protein samples were electrophoresed on 10% SDS-polyacrylamide gel before being transferred to PVDF membranes (Millipore, Bedford, MA, USA). The membranes were blocked with 5% non-fat milk at room temperature for 1 hour, followed by overnight incubation at 4°C with primary antibodies. Subsequently, the membranes were washed with TBS-Tween and then incubated with the appropriate HRP-conjugated secondary antibodies for 1 hour at room temperature. Finally, protein signals were visualized using a Bio-Rad system and electrochemical luminescence reagent (ECL) (Millipore, Bedford, MA, USA).

We received the following antibodies (NFATc1 (TD6446), c-fos (T56596), Cathepsin K (TD6614), MMP9 (TA5228), Parkin (T56641), DRP1 (TD7037), MFN2 (T56638), FIS1 (TD12005)) from Abmart (Shanghai, China), internal reference antibody (GAPDH (60004-1-Ig)) from Proteintech Group (Rosemont, IL, USA), and corresponding secondary antibodies from Jackson ImmunoResearch Inc. (West Grove, PA, USA).

### Cell migration assay

Following the previously described method [15], total number of 4 × 10^4^ BMMs were seeded on the transwell system (24-well insert, Corning, Tewksbury, MA, USA). In transwell system, BMMs were incubated for 12 h in the presence of CL before exposing to the bone marrow macrophage complete media, which was added as chemotactic agent in the bottom chamber. The upper surface of the membrane was swabbed with a cotton swab to remove non-migratory cells, while the cells on the lower surface were fixed and stained with a 1% crystal violet solution. Quantification was performed by counting the number of stained cells.

### Determination of mitochondrial membrane potential

Following the previously described method [16], BMMs were cultured with osteoclast induction media. During osteoclast differentiation, DMSO or CL was supplemented in osteoclast induction media. As directed by the manufacturer, the mitochondrial membrane potential of cells cultured for 3 days was measured by the JC-1 staining solution (Cayman Chemical, Ann Arbor, MI, USA).

### Transmission electron microscopy (TEM)

Following the previously described method [16], BMMs were cultured with osteoclast induction media. During osteoclast differentiation, DMSO or CL was supplemented in osteoclast induction media. On the third day, the cells were fixed with 2.5% glutaraldehyde, postfixed with 1% osmium tetroxide, dehydrated using an ethanol series, and finally embedded in resin. For TEM (HITACHI HT7700) observation of the embedded samples, the sections were cut to a thickness of 60 nm and stained with uranyl acetate and lead citrate. Mitochondria images from 15 cells were analyzed using ImageJ. Mitochondrial number was counted by Analyze Particles. Cristae density was estimated by the inner/outer mitochondrial membrane perimeter ratio.

### Murine model

The whole process of animal experiments was executed under supervision of the Animal Use and Care Committee of School of Medicine, Tongji University and following the guidelines of the NIH “Principles of Laboratory Animal Care” (1996 Revised Version). The specified-pathogen-free animal care facilities of Tongji University were used to maintain the wild-type C57/BL6 female mice (12 weeks old) purchased from SLAC Laboratory Animal Co. Ltd (Shanghai, China). In addition to feeding sterile chow and water, all mice were subjected to constant conditions of temperature (25 degrees centigrade), humidity (60 percent humidity), and illumination (12:12 cycles of light and dark). After adaption, mice were randomly divided into three groups (*n* = 6), one group with sham surgery (Sham), one group with bilateral ovariectomies (OVX), and the other with bilateral ovariectomies treated by CL. CL is administered at a dose of 30 mg/kg/d based on previous studies demonstrating no toxicity of CL in mice [18]. Following four weeks of drug administration, all mice were sacrificed and their orbital blood was collected along with their femurs and tibias.

### Bone tissue sample analysis

Following the removal of soft tissue and fixation, the femoral specimens underwent scanning and analysis using a micro-computed tomography system (SKYSCAN 1276, Bruker, German) with 10.5 μm resolution, 100 kV source voltage, and 98 mA source current. Based on our previous findings, we designed a protocol for μ-CT and histomorphometric analysis [19]. Specifically, we chose the area at 0.5 mm beneath subchondral bone for histomorphometric analysis. In addition, reconstruction of the femur in three dimensions, and measurement of bone volume/tissue volume (BV/TV), trabecular number (Tb.N), trabecular thickness (Tb.Th), and trabecular separation (Tb.Sp) were performed. For the tissue staining, the femurs were decalcified, embedded in paraffin, and cut into 5 μm sections.

### Statistical analysis

In this study, a minimum of three replicates were conducted in each experiment and the data was expressed as mean ± SD. In cases where there were more than two groups, the significant differences were determined by one-way ANOVA and Tukey–Kramer honest significant difference (HSD) test. In cases where there were two groups, the significant differences were determined by unpaired *t*-tests. Differences statistically significant were defined as *p* < 0.05.

## RESULTS

### CL inhibits osteoclast differentiation

**Figure 1:**
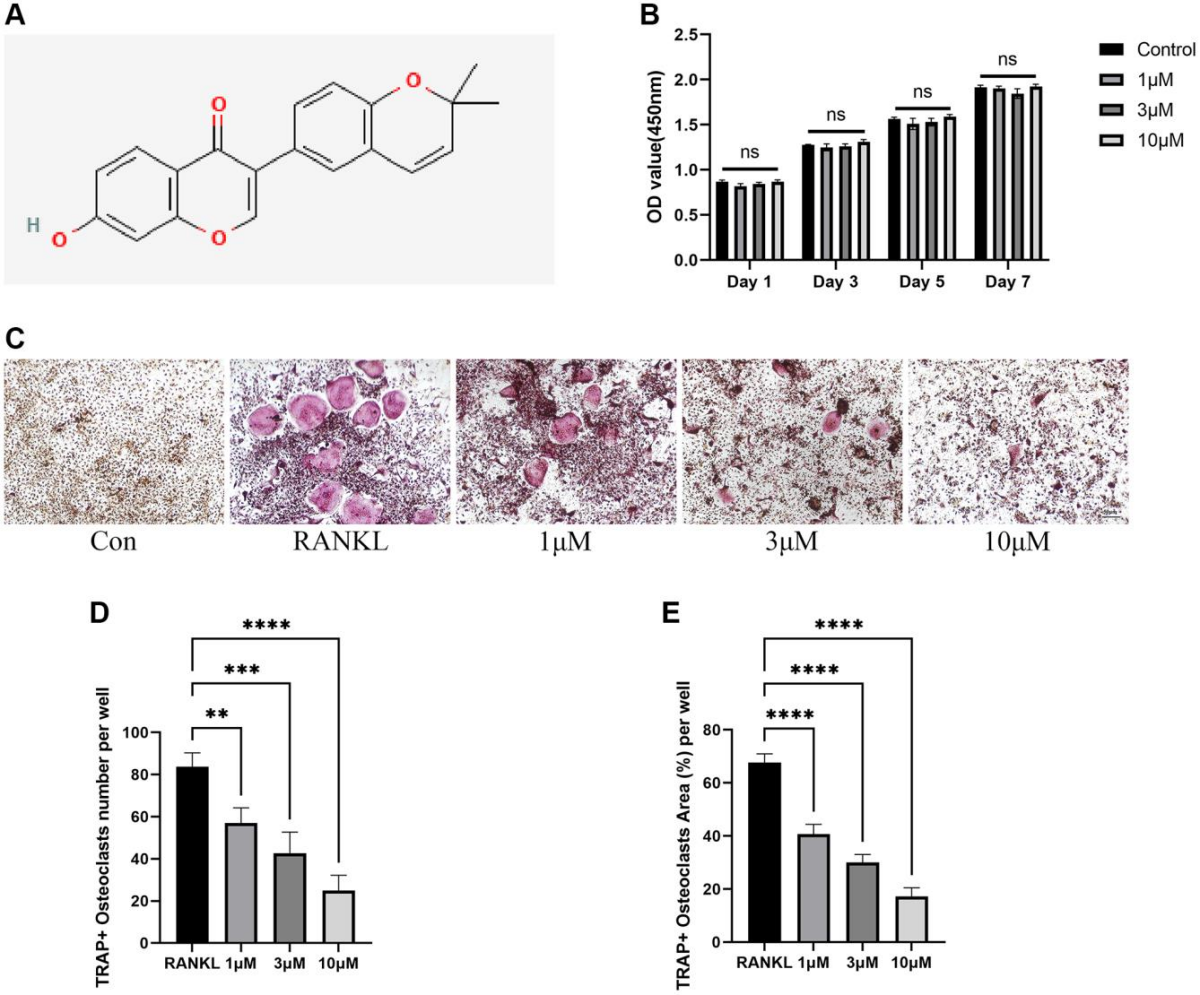
**Inhibition of RANKL-induced osteoclast differentiation by CL.** (**A**) Corylin structure chemically. (**B**) Cell viability of CL on BMMs at day 1, day 3, day 5, and day 7. (**C**) The TRAP staining image from BMMs treated with CL under osteoclast induction condition. (**D**, **E**) The number and area quantifying of mature osteoclast. The ns, not statistically significant; ^**^P < 0.01; ^***^P < 0.001; ^****^P < 0.0001.

To begin with, the potential toxicity of CL on BMMs was examined through CCK-8 assay and the results showed that BMMs viability were not affected by CL after being cultured with indicated doses for 1 day, 3 days, 5 days, and 7 days (Figure 1A, 1B). Then, we investigated how CL affects osteoclast differentiation by TRAP staining and we found the osteoclast formation was suppressed when the CL was supplemented in osteoclast induction media (Figure 1C–1E). Furthermore, the effect of CL inhibiting osteoclast differentiation is dose-dependent.

### CL suppresses osteoclast function

**Figure 2:**
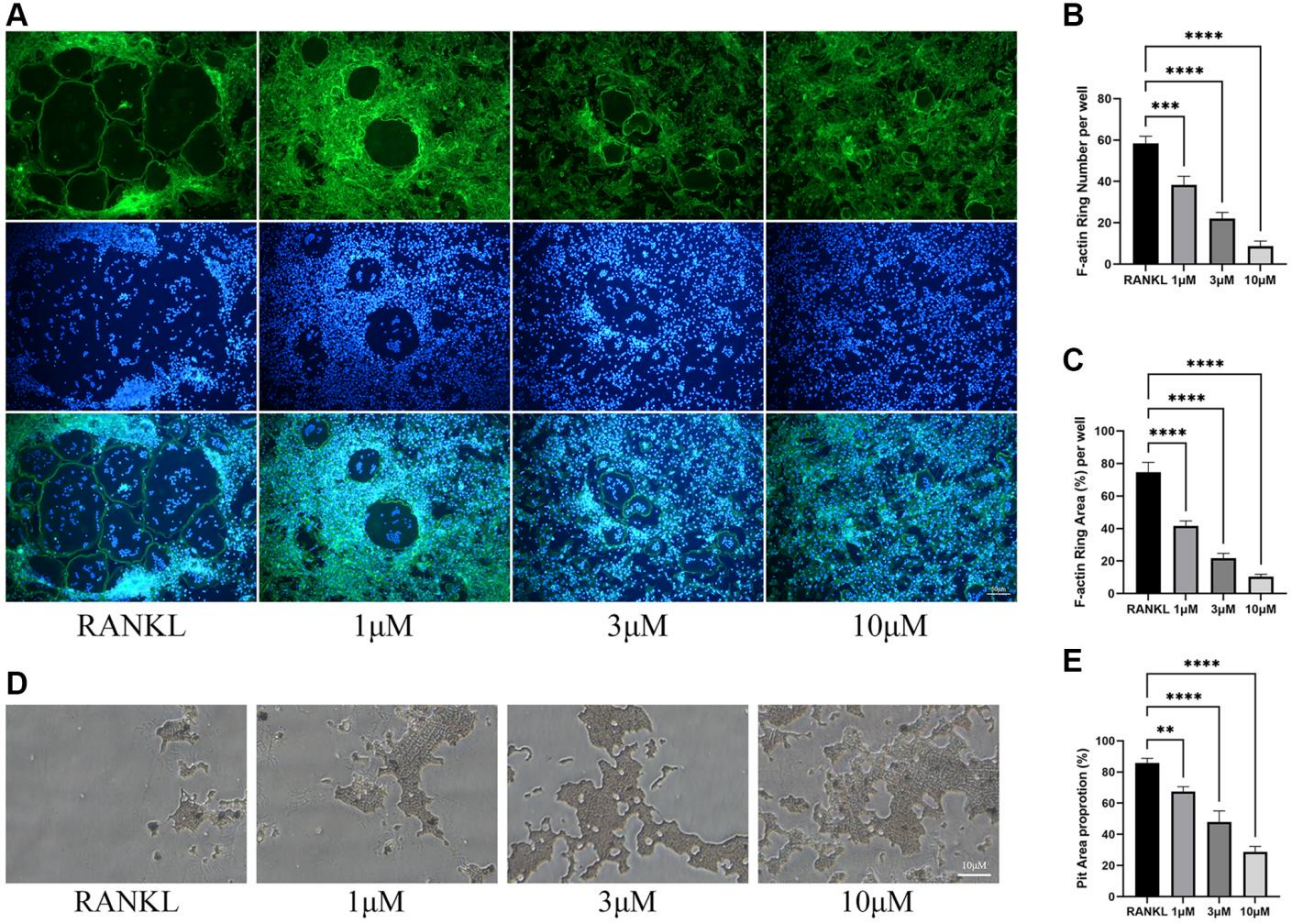
**Inhibition of osteoclast function by CL.** (**A**) The F-actin ring image from BMMs treated with CL under osteoclast induction condition. (**B**, **C**) The number and area quantifying of F-actin ring. (**D**) The bone resorption pits image form osteoclast treated with CL. (**E**) The area quantifying of bone resorption pits. ^**^P < 0.01; ^***^P < 0.001; ^****^P < 0.0001.

As osteoclast differentiation is inhibited by CL, we wondered whether the bone resorptive capacity of osteoclast would also be inhibited. During osteoclast differentiation in bone plate, CL could significantly reduce the formation of F-actin ring (Figure 2A–2C), which is commonly used as an indicator of its resorptive activity. Furthermore, the resorption pit caused by osteoclasts was lessened when formation osteoclasts were cultured with CL (Figure 2D, 2E).

### CL suppresses osteoclast-specific protein and gene expression

**Figure 3:**
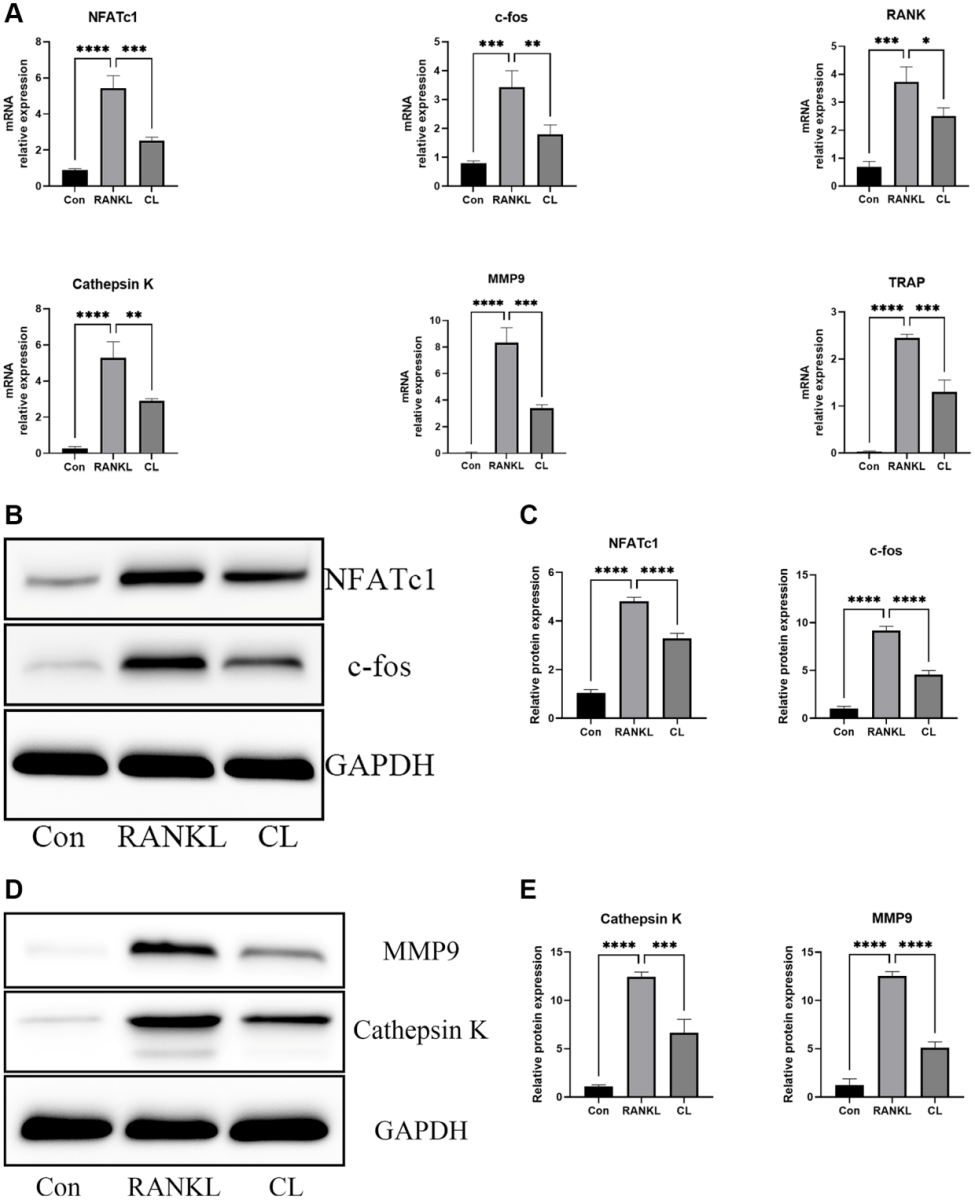
**Inhibition of RANKL-induced NFATc1 and c-fos transcription and downstream related genes by CL.** BMMs were treated with CL in osteoclast induction medium for 3 days. (**A**) The analysis of the relative mRNA levels of NFATc1, c-fos, RANK, Cathepsin K, MMP9, and TRAP. (**B**) The protein band image of NFATc1 and c-fos. (**C**) The expression quantifying of NFATc1 and c-fos. (**D**) The protein band image of Cathepsin K and MMP9. (**E**) The expression quantifying of Cathepsin K and MMP9. ^*^P < 0.05; ^**^P < 0.01; ^***^P < 0.001; ^****^P < 0.0001.

In order to further validate the results of CL inhibiting osteoclast differentiation and function at the transcription and translation level, a set of genes specifically involved in osteoclast differentiation (NFATc1, c-fos, RANK) and bone resorption (Cathepsin K, MMP9, and TRAP) were selected. In normalized data, it was found that CL markedly attenuated RANKL-stimulated expression of osteoclast-related genes (Figure 3A). At protein level, western blot analyses revealed the reduced proteins of NFATc1, c-fos, MMP9, and Cathepsin K in response to RANKL stimulation, which is consistent with the mRNA results (Figure 3B–3E).

### CL suppresses migration and fusion of pre-osteoclast

**Figure 4:**
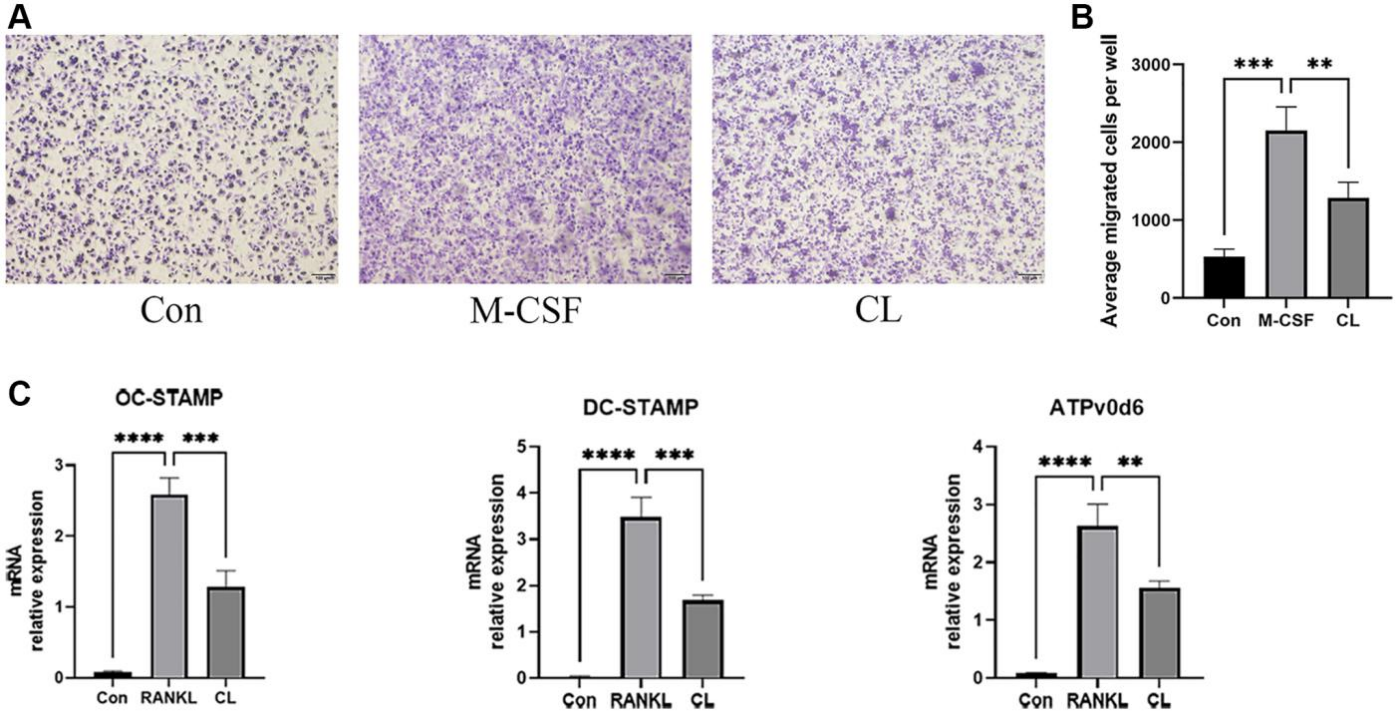
**Inhibition of pre-osteoclast migration and fusion by CL.** (**A**) The crystal violet staining image from pre-osteoclast treated with CL. (**B**) The number quantifying of migrated cells. (**C**) The analysis of the relative mRNA levels of OC-STAMP, DC-STAMP, and ATPv0d6. ^**^P < 0.01; ^***^P < 0.001; ^****^P < 0.0001.

Osteoclast development is a complex multi-step process involving triggering the differentiation of bone marrow macrophages into pre-osteoclasts, the migration of pre-osteoclasts, and the fusion of pre-osteoclasts into mature osteoclasts. During osteoclast development, the treatment of CL significantly inhibited migration of pre-osteoclasts induced by M-CSF in the migration assay (Figure 4A, 4B). In addition, the results of qRT-PCR revealed that the RANKL-induced expression of genes responsible for osteoclast fusion was suppressed by CL (Figure 4C).

### CL alleviates mitochondrial mass and function

**Figure 5:**
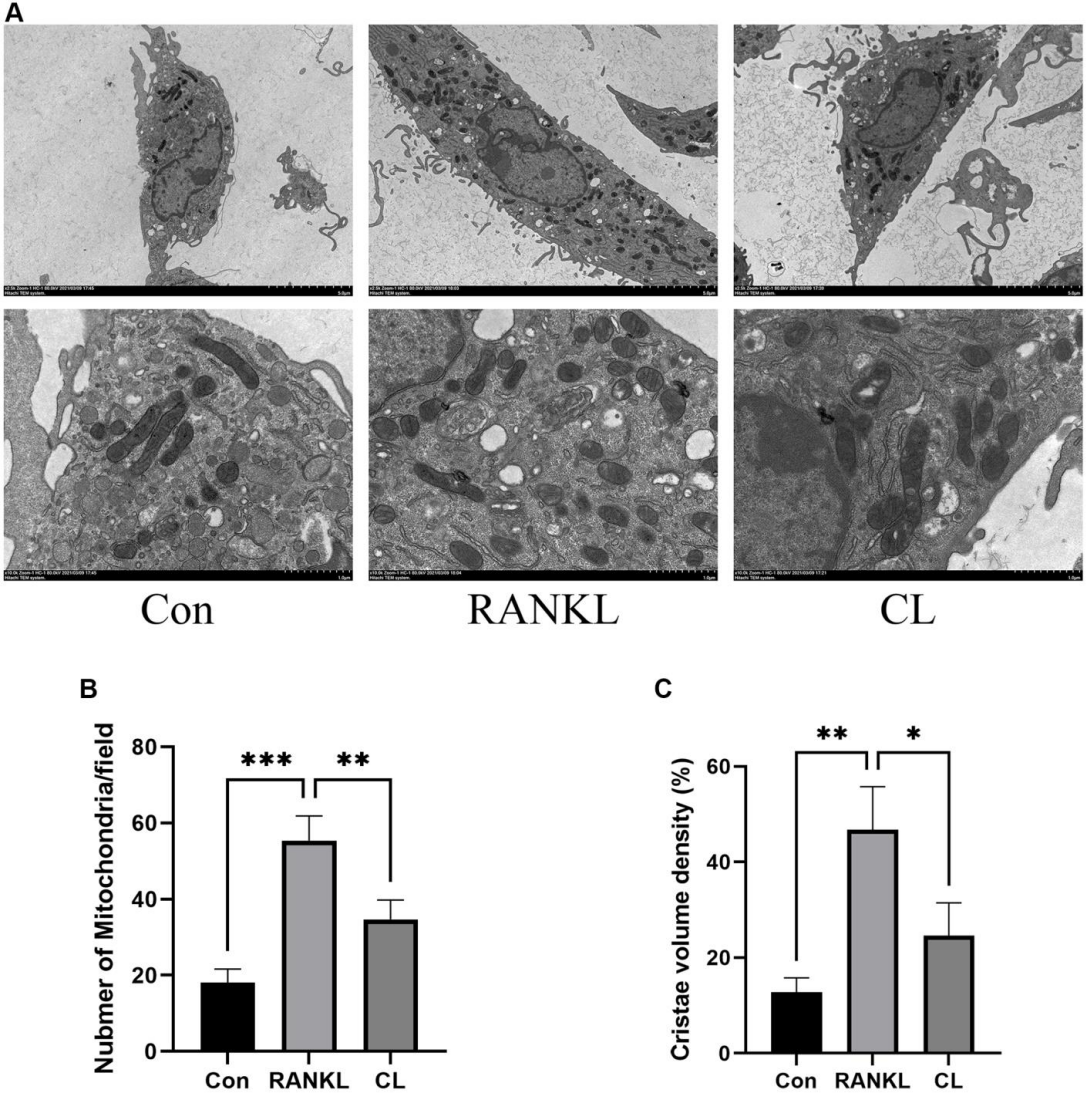
**Inhibition of mitochondrial number and mass by CL.** (**A**) The mitochondrial image from BMMs treated with CL under osteoclast induction condition. (**B**, **C**) The number and cristae density quantifying of mitochondria. ^*^P < 0.05; ^**^P < 0.01; ^***^P < 0.001.

**Figure 6:**
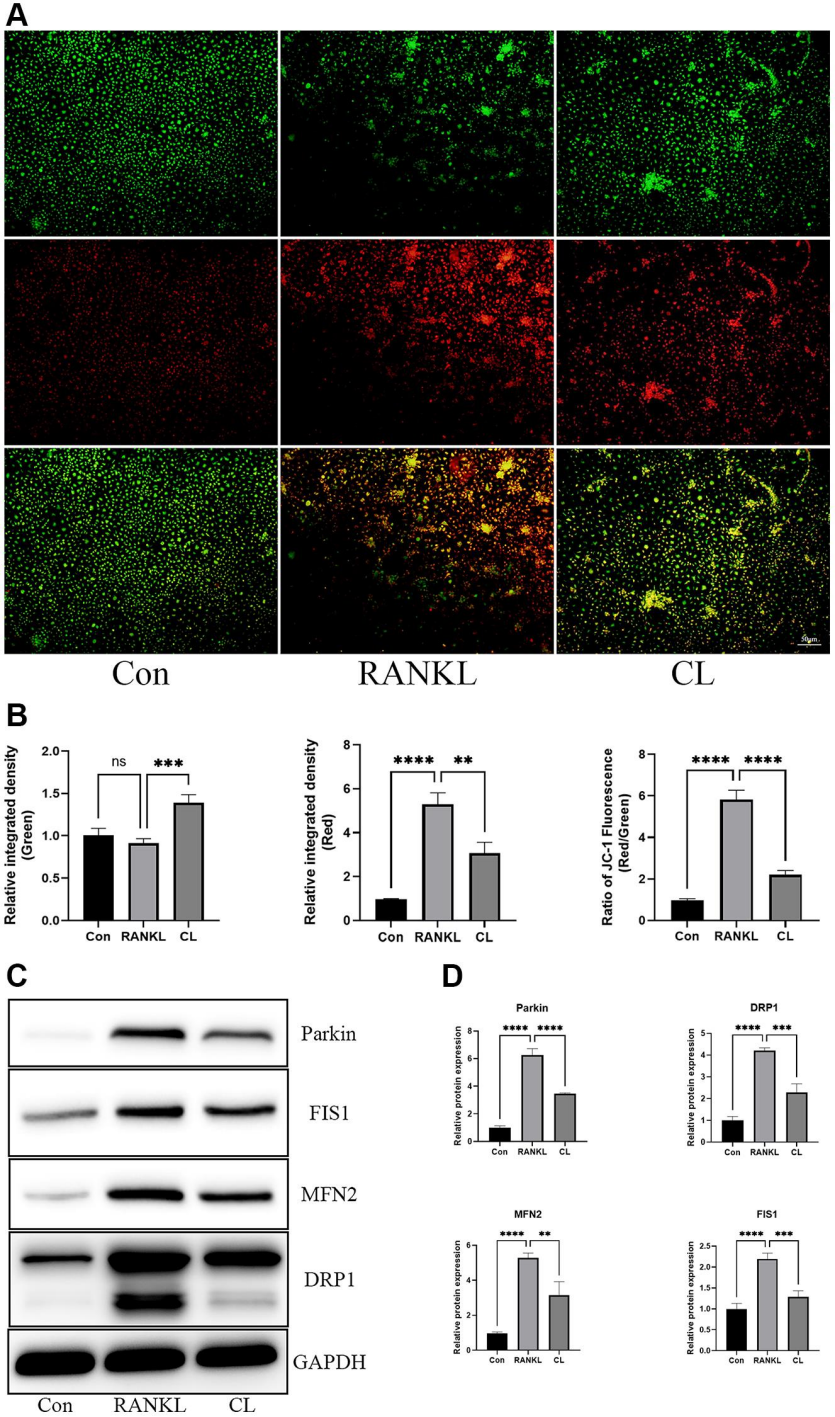
**Inhibition of mitochondrial function by CL.** (**A**) The JC-1 staining image from BMMs treated with CL under osteoclast induction condition. (**B**) The membrane potential quantifying of mitochondria. (**C**) The protein band image of Parkin, DRP1, FIS1, and MFN2. (**D**) The expression quantifying of Parkin, DRP1, FIS1, and MFN2. The ns, not statistically significant; ^**^P < 0.01; ^***^P < 0.001; ^****^P < 0.0001.

It has been described previously that osteoclast differentiation and activation are energy-intensive processes, resulting in an increase in mitochondrial number and activity within the cell [20]. During osteoclast development, the TEM revealed that the number of mitochondria increased, while CL reduced it. In addition, through observing the ultrastructure of mitochondria, we found that the density of cristae was richer in response to RANKL stimulation, and CL could attenuate that density (Figure 5). For measuring the activity of mitochondrial functions, we detected the mitochondrial membrane potential by JC-1 staining and found that CL significantly reduced the mitochondrial membrane potential during osteoclast differentiation (Figure 6A, 6B). According to previous study, the mitochondrial function and morphology were influenced by the fusion and fission of mitochondria [16]. Thus, we further investigated the protein level involved in mitochondrial fusion and fission. Our results indicate that RANKL stimulated the protein level of Parkin, DRP1, MFN2, and FIS1, whereas CL inhibited elevated protein levels (Figure 6C, 6D).

### CL mitigates OVX-induced bone loss

**Figure 7:**
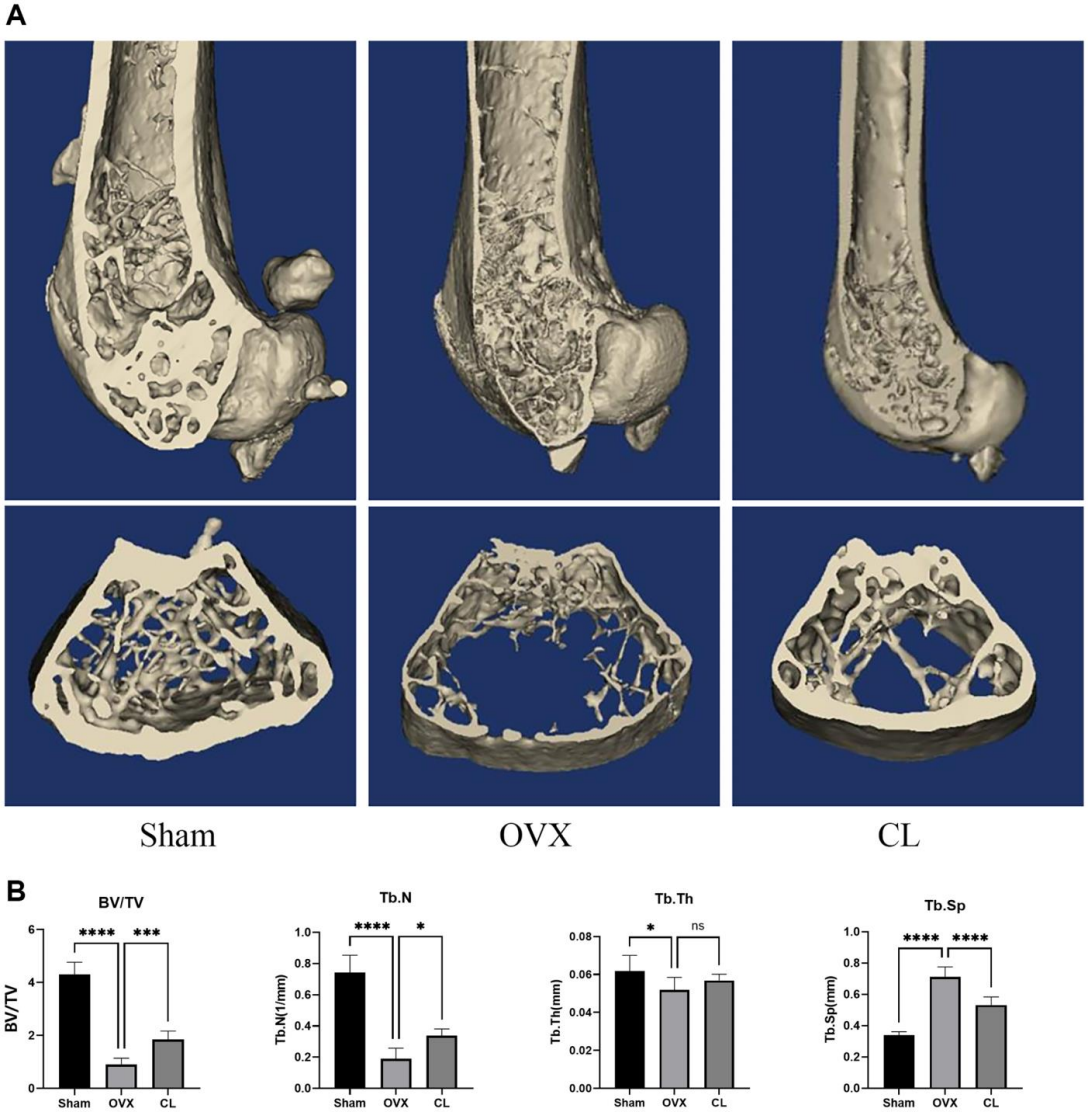
**Inhibition of OVX-induced bone loss by CL administration.** (**A**) The 3D reconstruction image from bone samples. (**B**) The quantifying analysis of bone quality through BV/TV, Tb.Th, Tb.N, and Tb.Sp. The ns, not statistically significant; ^*^P < 0.05; ^***^P < 0.001; ^****^P < 0.0001.

**Figure 8:**
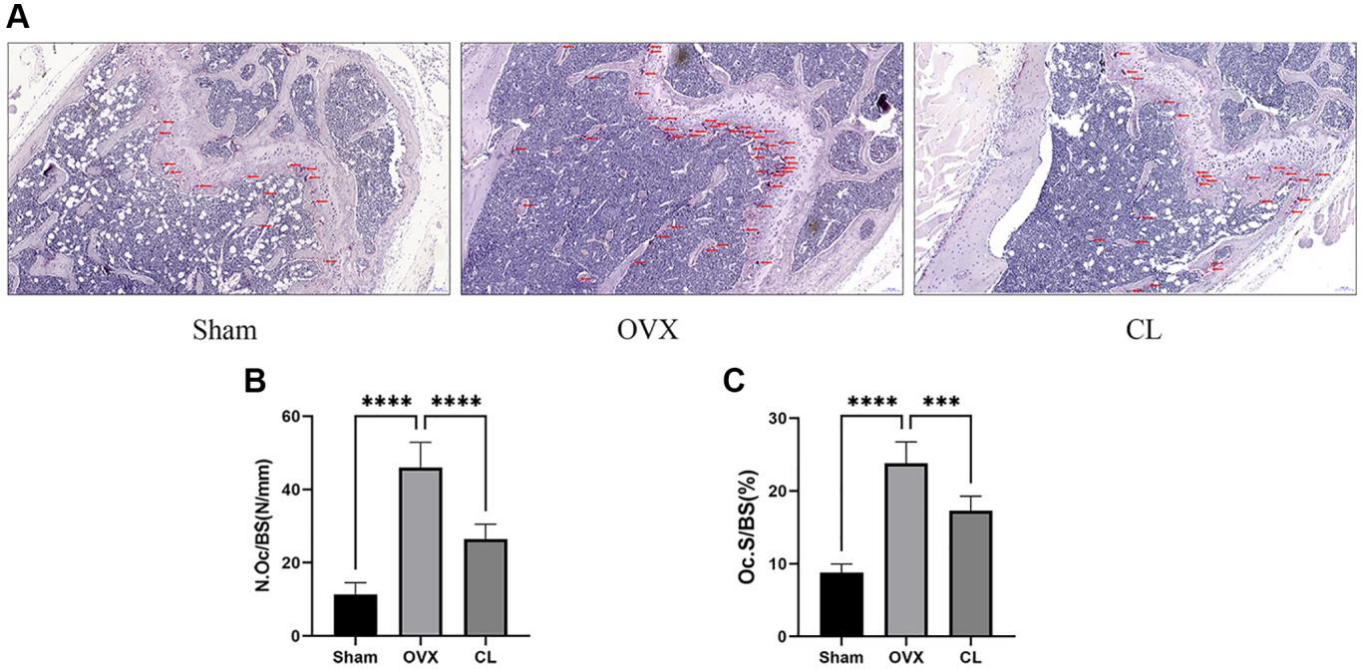
**Inhibition of osteoclast formation *in vivo* by CL administration.** (**A**) The TRAP staining image from bone samples. (**B**, **C**) The histomorphometric quantifying (N.Oc/BS and OcS/BS) of TRAP-positive osteoclasts. ^***^P < 0.001; ^****^P < 0.0001.

Our vitro study demonstrated that CL administration could modulate osteoclast differentiation and function, therefore we were interested in further examining its potential therapeutic effect for osteoporosis. Through scanning bone tissue, the CL treatment reversed the dramatically reduced bone mass induced by estrogen deficiency, as evidenced in decreased BV/TV, Tb.N, and elevated Tb.Sp (Figure 7). Following TRAP staining on bone sections, it was evident that the number and area of osteoclasts normalized to bone surfaces were both increased due to ovariectomy and administration of CL inhibited osteoclast differentiation *in vivo* (Figure 8). As a result of the data from the *in vivo* study, we conclude that ICT reduces bone loss caused by OVX via inhibiting osteoclasts formation.

## DISCUSSION

In the present study, we identify CL as a promising therapeutic agent for osteoporosis, substantiated by evidence from various perspectives. Initially, CL can restrain the formation of osteoclasts as well as actin rings. Besides, bone resorptive capacity of osteoclast was significantly suppressed in the presence of CL. In terms of molecular mechanism, CL possessed the ability to inhibit c-fos and NFATc1 expression and regulate cytoskeleton dynamics and mitochondrial mass and function. Finally, CL has been shown to reduce bone loss by preventing osteoclast formation in animal models.

Within the bone tissue, bone resorption is primarily facilitated by osteoclasts, which originate from precursor cells of the monocyte-macrophage lineage. Both transcription factor, c-fos and NFATc1 are indispensable regulatory factors for osteoclast formation. In previous studies, the mice with c-fos or NFATc1 mutant were found to have osteopetrotic phenotypes caused by a blockage in osteoclast differentiation [21, 22]. Transcription factor c-fos is initially activated during osteoclast differentiation and subsequently activates the NFATc1 promoter. This promotes the expression of NFATc1, which, in conjunction with c-fos, controls the expression of osteoclast-specific genes involved in matrix degradation [23]. Our study clearly demonstrates that CL significantly inhibited osteoclast-specific mRNA and protein expression.

During osteoclast development, BMMs initially differentiate into TRAP-positive monocyte with M-CSF and RANKL stimulation, which are also called pre- osteoclast, then the formed pre-osteoclasts migrate to bone surface and fuse with other pre-osteoclasts or formed osteoclasts to turn into functionally activated multinucleated osteoclast cells. As previously reported by the studies, blocking the migratory or fusion behaviors of pre-osteoclasts were also effective in reducing formation of mature osteoclast, thereby reducing pathologic bone loss in disease models [24–26]. Our study shows that the migration of pre-osteoclasts induced by M-CSF is inhibited in the presence of CL. Furthermore, CL treatment also attenuates the gene expression of DC-STAMP, OC-STAMP, and ATP6v0d2, responsible for regulating cell fusion.

It is always known that all cellular activities, such as gene transcription, protein synthesis, and cytoskeletal remodeling, are sustained by hydrolysis of adenosine triphosphate (ATP), synthesized by the mitochondria, which are the “powerhouse” of eukaryotic cells [16]. The process of osteoclast differentiation is known to be energetically demanding, as it relies on high metabolic activity. Accumulating evidence supports the pivotal role of mitochondria in intracellular signaling pathways governing osteoclast differentiation and maturation [16, 27]. Lemma and his colleague conducted a study where they observed an increase in mitochondrial mass during osteoclast differentiation, and through ultrastructural analysis, they found that the cristae structure in mature osteoclasts is more abundant [27]. In our study, we observe a simultaneous change in mitochondrial features throughout osteoclast differentiation. Furthermore, CL treatment leads to decreased mitochondrial mass and cristae density. Moreover, maintaining optimal levels of mitochondrial membrane potential is crucial for ATP production and serves as an indicator of overall mitochondrial function [28]. To investigate the impact of CL on mitochondrial membrane potential during osteoclast differentiation, we examined the effect of CL supplementation on the enhanced mitochondrial membrane potential induced by RANKL. Our findings reveal a weakening of the enhanced mitochondrial membrane potential following CL supplementation. In order to maintain the integrity of mitochondrial morphology, membrane potential, and function, it is crucial to maintain a balance between fusion and fission processes to eliminate damaged mitochondria [29]. Within mitochondria, DRP1 and Fission protein 1 (FIS1) plays a crucial role in mediating mitochondrial fission, leading to subsequent degradation of damaged mitochondria through both Parkin-dependent and Parkin-independent mitophagy pathways. In contrast, the fusion of two adjacent mitochondria occurs, resulting in the formation of an elongated mitochondrion facilitated by Mitofusin 2 (MFN2). The induction of RANKL leads to the stimulation of mitochondrial fusion and division in the process of osteoclast differentiation, while CL treatment significantly impacts these processes. The results suggest that osteoclast differentiation is associated with increased mitochondrial number and mass, elevated mitochondrial membrane potential, and activated mitochondrial process of fusion and fission, all of which are attenuated when CL is added.

Furthermore, it is crucial to recognize a notable limitation that cannot be overlooked in our study. While the inhibitory effect and underlying mechanism of CL on osteoclast differentiation have been extensively explored, the precise molecular target of CL’s action within osteoclast differentiation remains unclear. Hence, conducting comprehensive additional research is essential to identify the binding molecules of CL, thus advancing our understanding of its involvement in osteoclast differentiation.

According to our findings, CL can restrain osteoclast differentiation and prevent bone loss caused by estrogen deficiency through inhibiting vital transcription factor expression, restricting cytoskeletal remodeling, and affecting mitochondrial function and mass. Consequently, our study provides new insight into how CL may be used to treat osteoporosis and osteoclast-related bone metabolic diseases.
